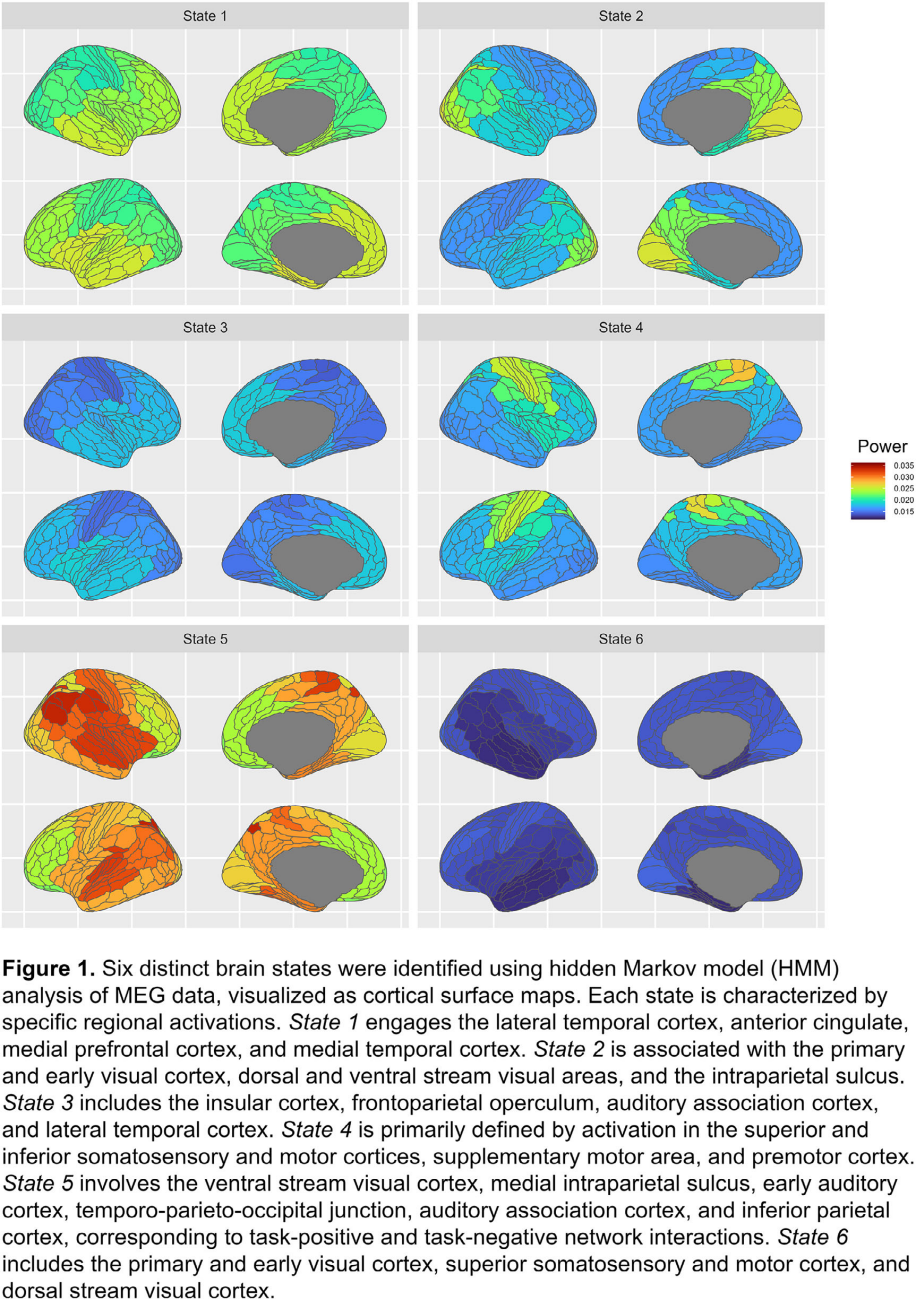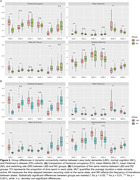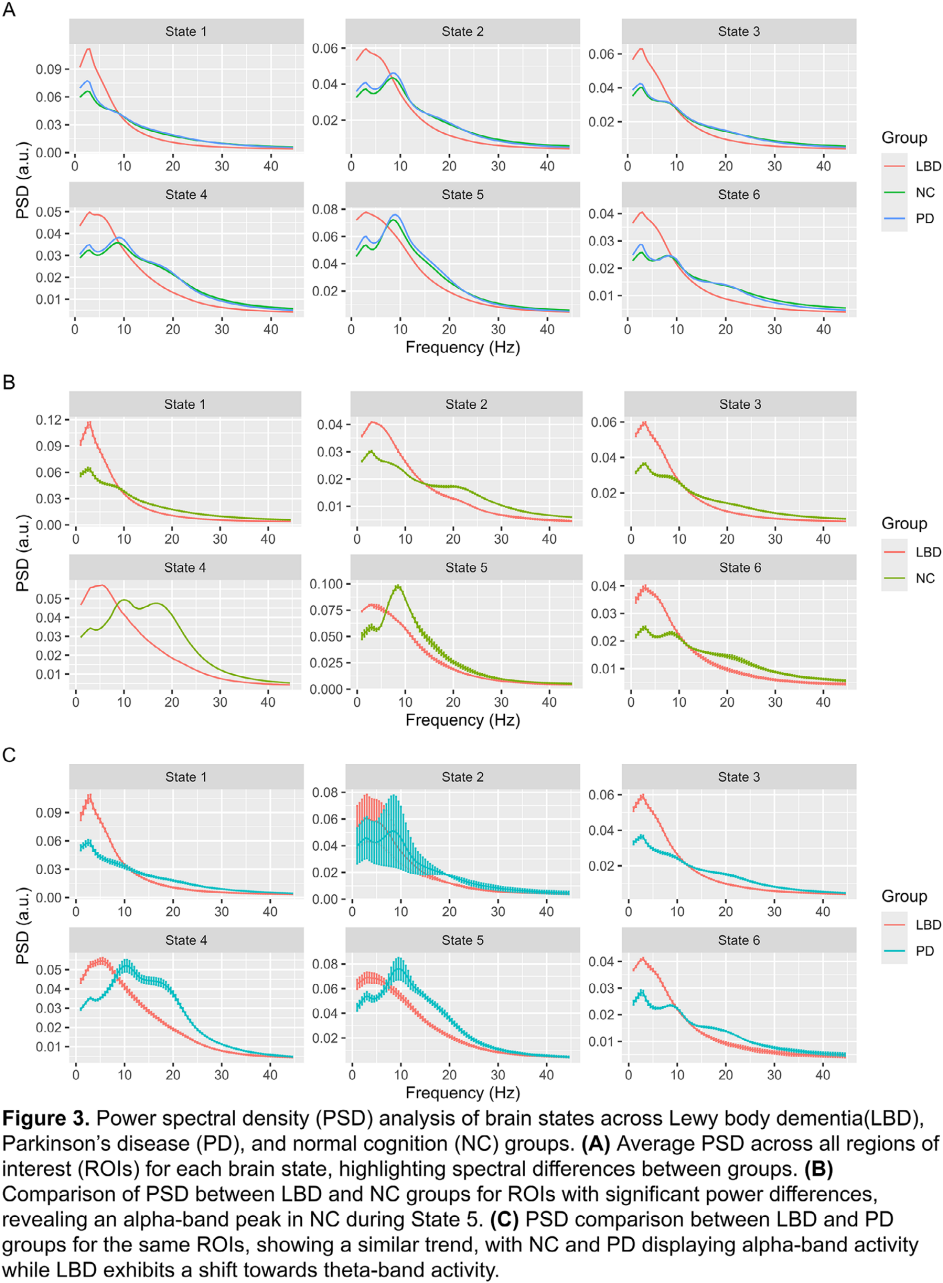# MEG Highlights Abnormal Neural Transitions in Lewy Body Dementia

**DOI:** 10.1002/alz70858_106084

**Published:** 2025-12-26

**Authors:** Hojjatollah Sadeqi, Zohreh Morshedizad, Babak Ahmadi, Rachael Burke, Melissa J. Armstrong, Abbas Babajani‐Feremi

**Affiliations:** ^1^ Fixel Institute for Neurological Diseases, Gainesville, FL, USA; ^2^ University of Florida, Gainesville, FL, USA; ^3^ Florida Alzheimer's Disease Research Center, Gainesville, FL, USA

## Abstract

**Background:**

Lewy body dementia (LBD) is the second most common degenerative dementia after Alzheimer's disease, yet its underlying neural mechanisms, particularly those contributing to cognitive fluctuations, remain poorly understood.

**Method:**

We analyzed resting‐state MEG data from 28 participants in three cohorts: (a) LBD (*n* = 7, age = 70.1 ± 5.7 years, 5 males), (b) Parkinson's disease (PD) without cognitive impairment (*n* = 8, age = 71.1 ± 7.6 years, 3 males), and (c) normal cognitive (NC) (*n* = 13; age = 72.3 ± 6.5 years, 5 males). A six‐state hidden Markov model (HMM) was applied to investigate brain state dynamics and cognitive fluctuations. Brain states were characterized by activation patterns across 52 regions of interest (ROIs). Four dynamic connectivity metrics were assessed: fractional occupancy (FO), mean lifetime (MLT), mean interval, and switching rate (SWR). Their correlations with the Clinician Assessment of Fluctuation (CAF) scale were also evaluated. Additionally, power spectral density (PSD) analysis was conducted to uncover neural signatures underlying cognitive variability in LBD.

**Result:**

Power maps for each brain state revealed distinct activation patterns (Figure 1). The most pronounced group differences were observed in States 1 and 5 (Figure 2). State 1, primarily involving the medial prefrontal and lateral temporal cortices, showed significantly prolonged occupancy (higher FO and MLT) in LBD patients compared to NC and PD groups (*p* < 0.001). Conversely, State 5, which engaged the right temporo‐parieto‐occipital regions, was less active in LBD. PSD analysis indicated slowing in LBD, with activity shifting from the alpha band in NC and PD groups to the theta band in LBD (Figure 3). Moreover, dynamic connectivity measures were significantly correlated with CAF across all states. Notably, FO in State 1 exhibited a strong positive correlation with CAF (*r* = 0.72, *p* < 10^‐24^), while FO in State 5 showed a strong negative correlation (*r* = ‐0.65, *p* < 10^‐18^).

**Conclusion:**

Our findings reveal distinct LBD‐specific spectral slowing and dynamic connectivity patterns, characterized by prolonged engagement in temporal‐prefrontal networks and reduced involvement in temporo‐occipito‐parietal regions. These alterations strongly correlate with cognitive fluctuations, suggesting potential biomarkers for LBD. Future research should explore targeting these abnormal state transitions to mitigate cognitive fluctuations.